# Integrative Analysis of Gut Microbiota and Fecal Metabolites in Rats after Prednisone Treatment

**DOI:** 10.1128/Spectrum.00650-21

**Published:** 2021-11-10

**Authors:** Jing Zhang, Dan Feng, Helen Ka-Wai Law, Ying Wu, Guang-hua Zhu, Wen-yan Huang, Yulin Kang

**Affiliations:** a Department of Nephrology and Rheumatology, Shanghai Children’s Hospital, Shanghai Jiao Tong University, Shanghai, China; b Department of Health Technology and Informatics, Faculty of Health and Social Science, The Hong Kong Polytechnic University, Hunghom, Hong Kong, China; Lerner Research Institute

**Keywords:** prednisone, gut microbiota, fecal metabolite, short-chain fatty acid

## Abstract

Prednisone (PRED) is a synthetic glucocorticoid (GC) widely used in immune-mediated diseases for its immunosuppressive and anti-inflammatory properties. The effects of GC are achieved by genomic and nongenomic mechanisms. However, the nongenomic effects are largely unknown. Thus, we aimed to investigate how long-term prednisone therapy changes the composition of the gut microbiota and fecal metabolites in rats. Male Sprague-Dawley rats were randomly assigned to a control (CON) group and a PRED group, which received prednisone treatment daily for 6 weeks by gavage. The V3 to V4 regions of bacterial 16S rRNA genes were amplified and sequenced after the total bacterial DNA was extracted from fecal samples. The alpha and beta diversities were calculated. The compositional alteration of the gut microbiota at different taxonomic levels was analyzed using the Metastats method. Meanwhile, the fecal metabolites were quantitated in an ultra-performance liquid chromatography system. Similar microbial richness and diversity between the CON and PRED groups were indicated by the alpha diversity results. The gut microbial communities differed significantly between two groups. The relative abundances of the genera *Eisenbergiella*, *Alistipes*, and *Clostridium* XIVb decreased, whereas that of *Anaerobacterium* increased significantly in rats after the 6-week prednisone treatment. In total, 11 downregulated and 10 upregulated fecal metabolites were identified. Differential fecal metabolites were enriched in the pathways, including phenylalanine metabolism, butanoate metabolism, and propanoate metabolism. The lowered production of short-chain fatty acids was associated with the decreased relative abundance of the genera *Alistipes* and *Clostridium XIVb* and increased abundance of the genus *Anaerobacterium*. The composition of the gut microbiota and fecal metabolites was changed after long-term prednisone treatment. This may help us to understand the pharmacology of prednisone.

**IMPORTANCE** Prednisone is widely used in chronic glomerular diseases, immunological disorders, and rheumatic diseases for its anti-inflammatory and immunosuppressive properties. It is a synthetic glucocorticoid (GC) that shows therapeutic effects after conversion to prednisolone by the liver. Prolonged GC therapy causes anti-inflammatory effects; it also results in a variety of adverse events, including obesity, hypertension, psychiatric symptoms, and dyslipidemia. The therapeutic effects and adverse events of GCs may be associated with changes in the gut microbiota, as the host might be affected by the metabolites generated by the altered gut microbes. Thus, we investigated how long-term prednisone therapy changed the composition of the gut microbiota and fecal metabolites in rats. This study may shed new light on the pharmacology of prednisone.

## INTRODUCTION

Prednisone is widely used in treating chronic glomerular diseases, immunological disorders, and rheumatic diseases for its anti-inflammatory and immunosuppressive properties. It is a synthetic glucocorticoid (GC) that shows therapeutic effects after conversion to prednisolone by the liver. The effects of GC are achieved by genomic and nongenomic mechanisms (1). When GC diffuses into the cell membrane, it binds to the GC receptor (GR) in the cytoplasm. Thereafter, the GC/GR complex is translocated to the nucleus and regulates gene expression through this so-called genomic mechanism. GCs have been shown to downregulate the expression of proinflammatory genes while inhibiting anti-inflammatory genes (2). For the nongenomic response, the related mechanisms are largely undefined. Some studies have suggested that GC binds directly to the membrane-bound GRs to inhibit the inflammatory response (3). Therefore, the nongenomic effects of GC treatment are worthy of further exploration.

Recently, the gut microbiota has become recognized as an immunological organ. Trillions of microbes colonize the intestinal tract. Mutualistic symbiosis has formed after millions of years of coevolution between the gut microbes and the host. The hosts provide a hospitable and nutritious environment for gut microbes, which in turn help to digest food, produce beneficial substances, and protect the host against pathogens. Gut microorganisms participate in the development, maturation, and function of the host immune system (4). Commensal gut microbes are essential for differentiation of some lymphocyte subsets. For instance, *Clostridia* and *Bacteroides* strains are inducers of regulatory T (Treg) cells (5, 6). Dysbiosis of the gut flora is attributed to immunological diseases, such as rheumatoid arthritis, systemic lupus erythematosus, and inflammatory bowel disease (4). It was also reported that germfree rodents are characterized by immunologic defects, such as decreased lamina propria CD4^+^ T cells and IgA-secreting plasma cells (7). Meanwhile, many factors affect the ecosystem of the gut microbiota, including medications, aging, diseases, gender, ethics, and food intake (8, 9). Studying the gut microbiota helps us understand the pharmacology of medications and the pathogenesis of diseases.

Gut microbiota produce diet-dependent and independent metabolites, some of which are indispensable for host immunity (10). The main source of fecal metabolites originates from the digestion and fermentation of dietary constituents. Meanwhile, gut microbes can also synthesize metabolites such as polysaccharide A. Short-chain fatty acids (SCFAs) are well-known metabolites generated by the fermentation of undigested polysaccharides by *Firmicutes* and *Bacteroidetes* strains (11, 12). Differentiation of Treg cells could be induced in the colon by the SCFA butyrate. Other bioactive microbial metabolites, such as indole and secondary bile acids, also affect the maturation and function of immune cells (10). Thus, it is suggested that fecal metabolites bridge the gut microbiota and host immunity.

Long-term GC treatment is commonly recommended in chronic immune-mediated diseases. Even though prolonged GC therapy exhibits anti-inflammatory effects, it results in a variety of adverse events, including obesity, hypertension, psychiatric symptoms, and dyslipidemia (1). The therapeutic effects and adverse events of GCs may also be associated with changes in the gut microbiota, as the host might be affected by metabolites generated by the altered gut microbes. Dysbiosis was shown to present in individuals with GC-induced obesity (13). GCs also change the composition of the gut microbiota in mice and birds (14, 15). A 4-week corticosterone treatment decreased the relative abundance of *Firmicutes* but augmented that of *Bacteroidetes* in mice (16). However, it remains unclear how prolonged prednisone therapy changes the composition of the gut microbiota in rats, which are commonly used for renal experiments. Moreover, the composition of fecal metabolites after prednisone treatment is seldom reported.

In the present study, alterations in the gut microbiota and fecal metabolites in rats after long-term prednisone treatment were investigated. Furthermore, we analyzed the correlation between the gut microbiota and fecal metabolites. This may not only help us to understand the pharmacology of prednisone but may also reveal the pathogenesis of adverse events.

## RESULTS

### Altered gut microbiota in rats receiving long-term prednisone treatment.

**FIG 1:**
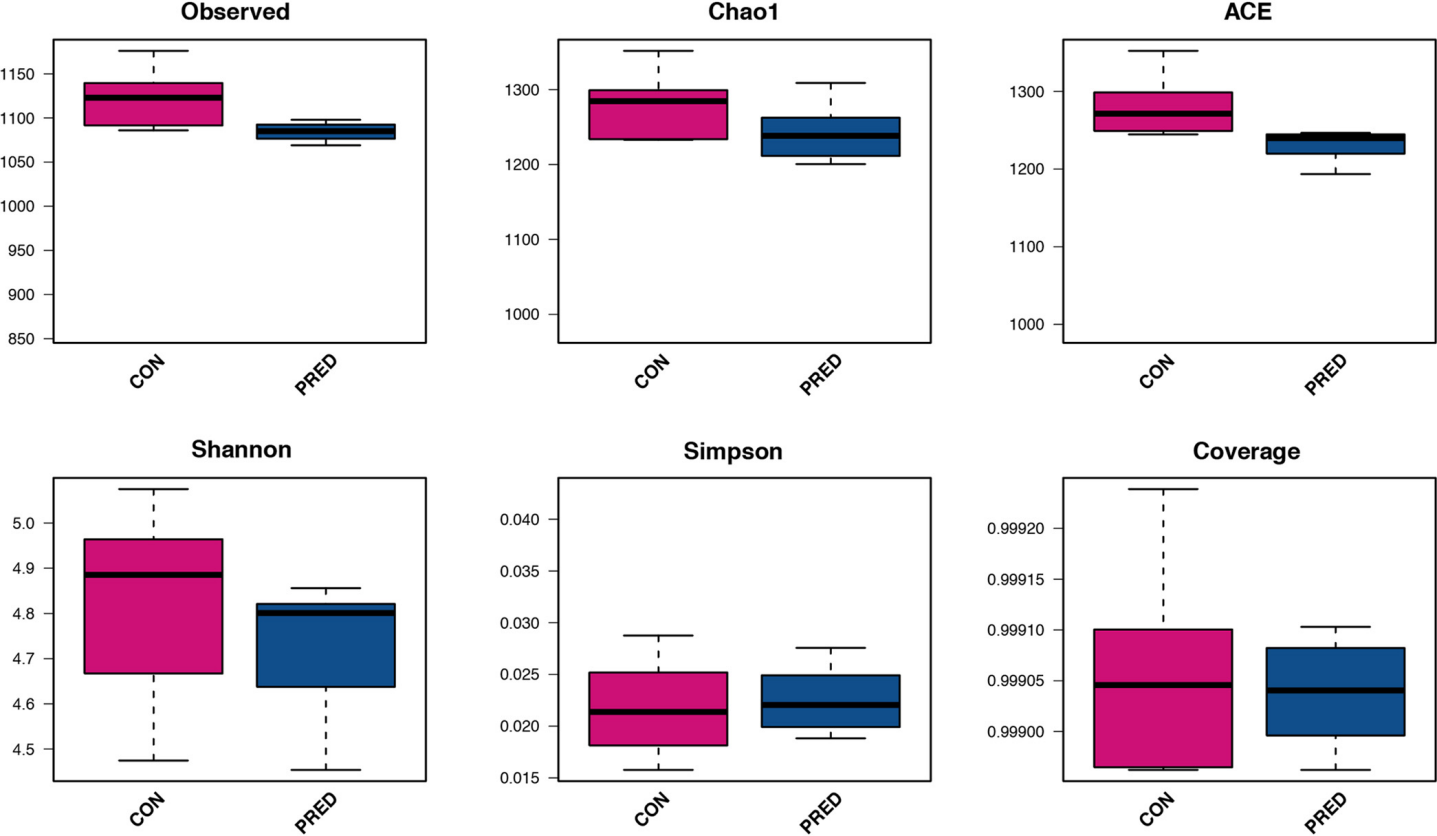
Diversity and richness of the gut microbiota in rats after a 6-week prednisone treatment. The observed OTUs and Chao1 and ACE indices represent the microbial richness, while the Shannon, Simpson, and Coverage indices indicate the microbial diversity. There were no significant differences in the microbial richness and diversity between the CON and PRED groups. *P > *0.05. Abbreviations: CON, control group. PRED, prednisone group.

**FIG 2:**
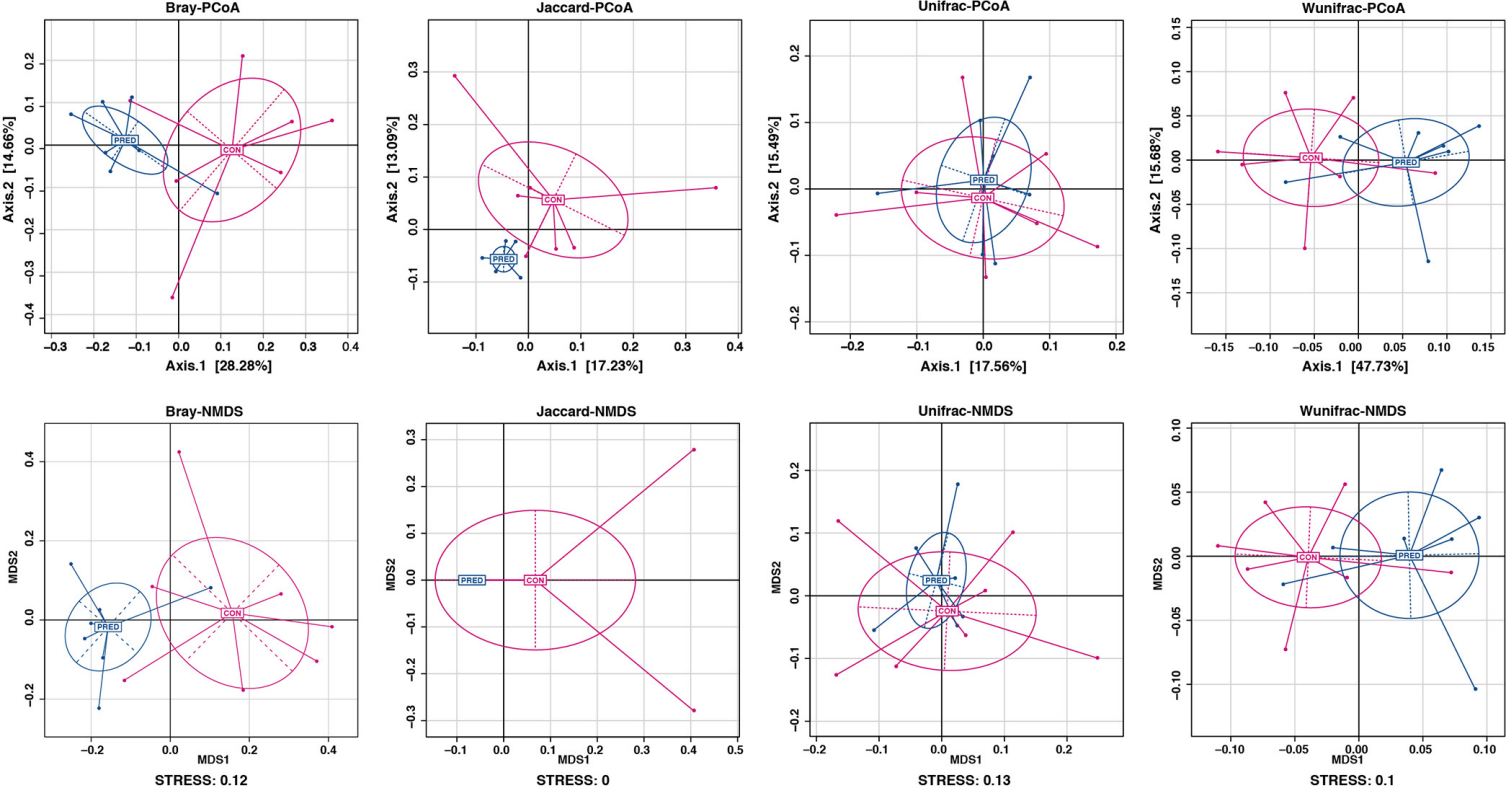
Principal-coordinate analysis and nonmetric multidimensional scaling profile of gut microbial diversity. The principal-coordinate analysis and nonmetric multidimensional scaling profile were based on the Bray-Curtis distance, Jaccard, and unweighted and weighted UniFrac metrics. They showed that the gut microbial community differs in rats with and without prednisone therapy. Abbreviations: PCoA, principal-coordinate analysis; NMDS, nonmetric multidimensional scaling; Bray, Bray-Curtis distance; unifrac, unweighted UniFrac metric; wunifrac, weighted UniFrac metric.

**FIG 3:**
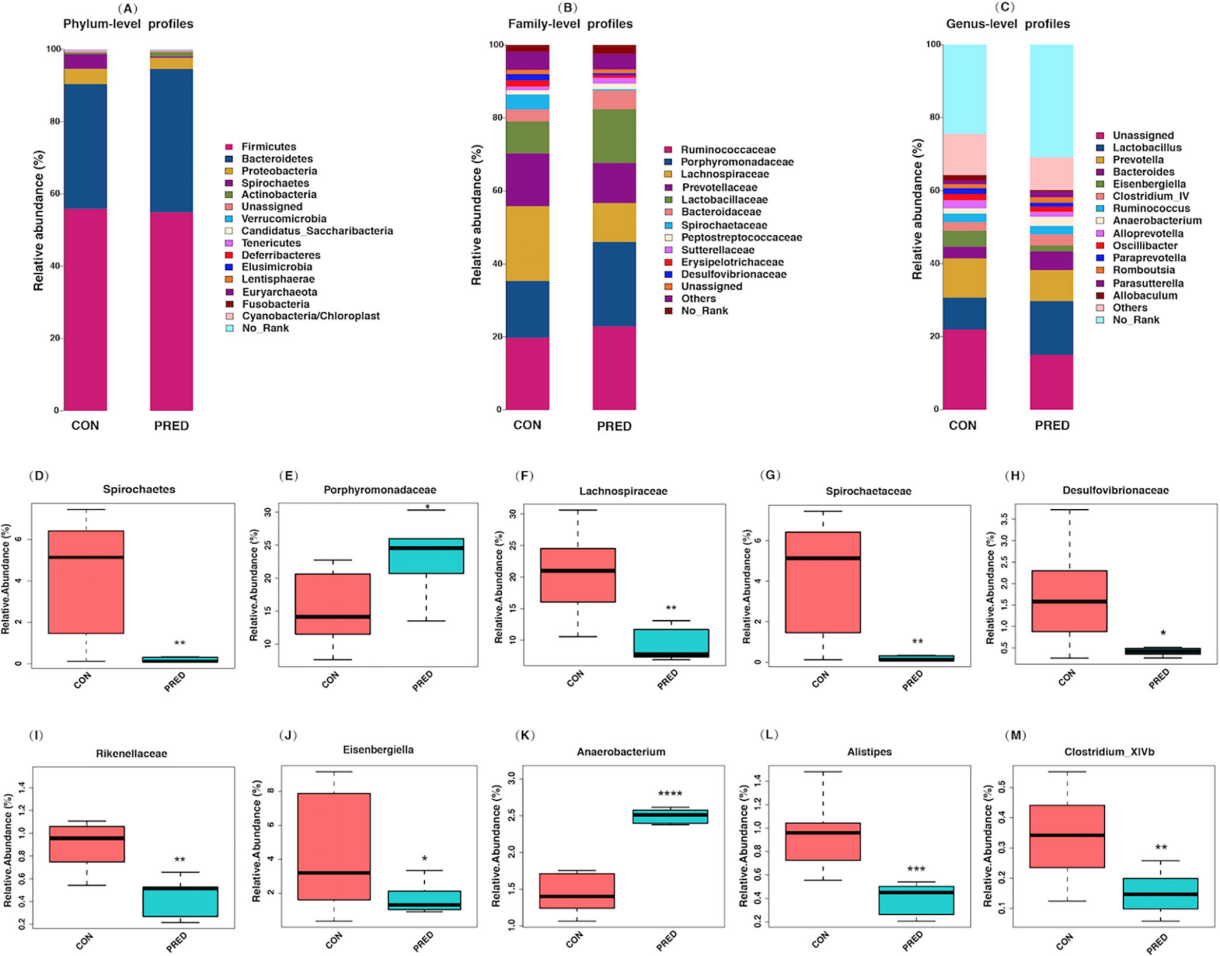
Compositional alteration of the gut microbiota at different taxonomic levels after prednisone treatment. Bar plots of the gut microbiota at the phylum (A), family (B), and genus (C) levels. They show that *Firmicutes*, *Bacteroidetes*, and *Proteobacteria* dominated the gut microbiota at the phylum level. *Ruminococcaceae*, *Porphyromonadaceae*, *Prevotellaceae*, *Lactobacillaceae*, and *Bacteroidaceae* are the major families of the fecal microbiota. In addition, *Lactobacillus*, *Prevotella*, *Bacteroides*, *Eisenbergiella*, and *Clostridium* XIVb are the major identified genera. The compositional alteration of the gut microbiota at different taxonomic levels was analyzed using the Metastats method. The relative abundance of the phylum *Spirochaetes* decreased after prednisone therapy (D). Increased relative abundance was observed for *Porphyromonadaceae* (E), while the abundance decreased for *Lachnospiraceae* (F), *Spirochaetaceae* (G), *Desulfovibrionaceae* (H), and *Rikenellaceae* (I) at the family level. The relative abundances of the genera *Eisenbergiella* (J), *Alistipes* (L), and *Clostridium* XIVb (M) decreased significantly, while that for *Anaerobacterium* increased (K) by comparison with the CON group. *, *P < *0.05; **, *P < *0.01; ***, P < 0.001; ****, P < 0.0001.

There were no significant differences in the body weight of either group after the 6-week prednisone treatment (Fig. S1). In total, 2,044 operational taxonomic units (OTUs) were detected (Text S1 in the supplemental material). The microbial richness was evaluated by analyzing the observed OTUs and the Chao1 and abundance-based coverage estimator (ACE) indices, while the microbial diversity was reflected by the Shannon, Simpson and Coverage indices. Similar microbial richness and diversity between the control (CON) and prednisone (PRED) groups are indicated in Fig. 1. Beta diversity analysis consisted of principal-coordinate analysis (PCoA) and nonmetric multidimensional scaling (NMDS), which were based on Bray-Curtis, Jaccard, and unweighted and weighted UniFrac metrics. Figure 2 shows the phylogenetic tree-based distances between individual fecal microbiota in a scatterplot. Plotting the results of the PCoA and NMDS revealed that the gut microbial community differs significantly in rats with and without prednisone therapy (Fig. 2). As shown in Fig. 3, *Firmicutes*, *Bacteroidetes*, and *Proteobacteria* dominated the gut microbiota at the phylum level. *Ruminococcaceae*, *Porphyromonadaceae*, *Prevotellaceae*, *Lactobacillaceae*, and *Bacteroidaceae* were the major families of fecal microbiota. *Lactobacillus*, *Prevotella*, *Bacteroides*, *Eisenbergiella*, and *Clostridium* XIVb were the major genera identified at the genus level. The compositional changes in the gut microbiota at different taxonomic levels were explored using the Metastats method. At the phylum level, the relative abundance of *Spirochaetes* decreased after prednisone therapy. Meanwhile, increased relative abundance was observed for *Porphyromonadaceae*, while relative abundance decreased for *Lachnospiraceae*, *Spirochaetaceae*, *Desulfovibrionaceae*, and *Rikenellaceae* at the family level. The PRED group showed a decreased relative abundance of fecal microbes at the genus level, including the genera *Eisenbergiella*, *Alistipes*, and *Clostridium* XIVb, whereas the abundance of *Anaerobacterium* increased significantly.

### Prednisone treatment changed the profile of fecal metabolites in rats.

**FIG 4:**
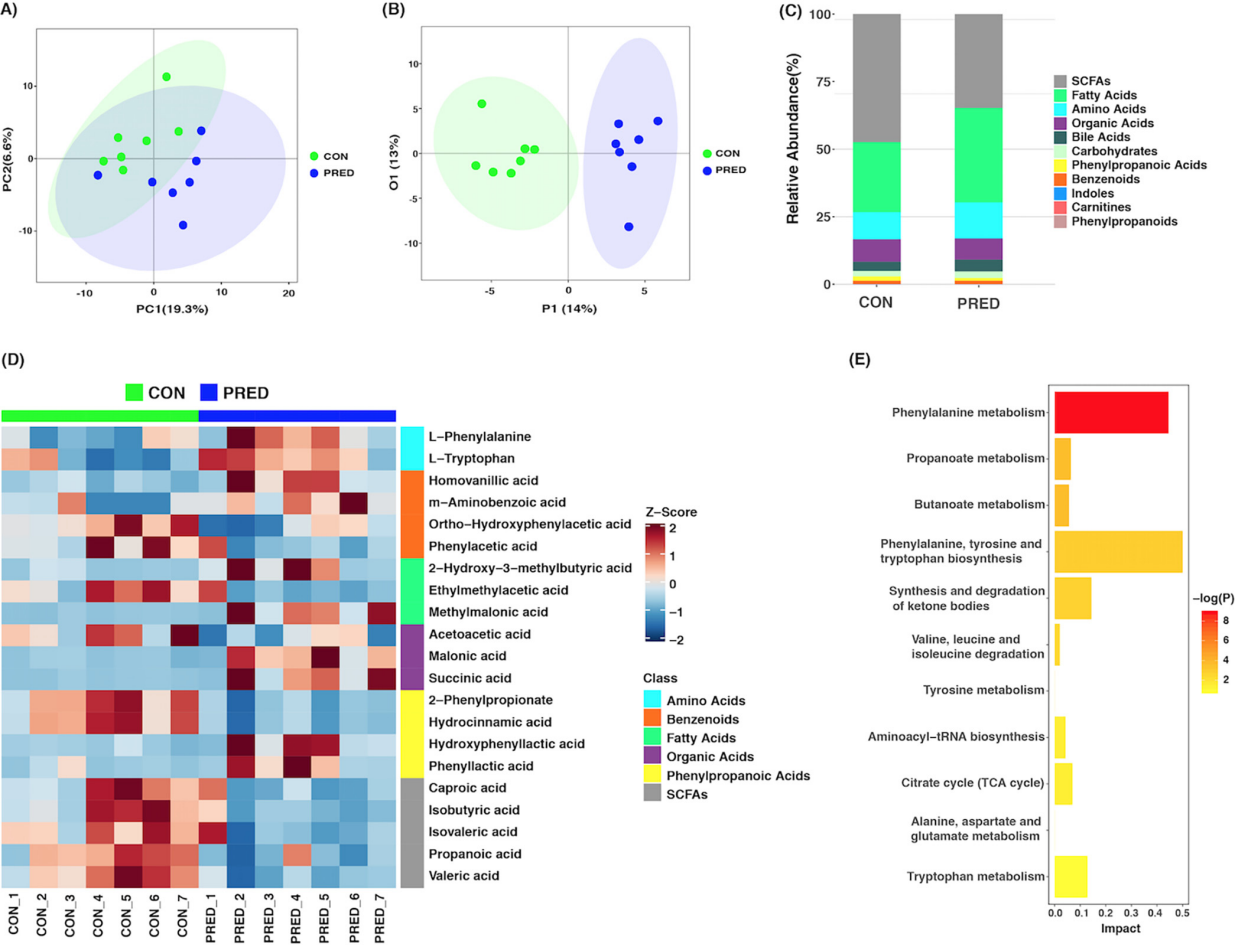
Fecal metabolomics was altered by the 6-week prednisone treatment. (A) Principal-component analysis (PCA) plot of fecal metabolites in rats with and without prednisone treatment. It shows that the overall fecal metabolite profiles differed in the two groups. (B) Score plot of orthogonal partial least-squares discriminant analysis (OPLS-DA). It reveals that the composition of fecal metabolites changed significantly after the 6-week prednisone treatment. (C) Bar plot of the identified fecal metabolites in the two groups. (D) Heatmap of differential fecal metabolites. The significantly changed fecal metabolites include SDFAs, fatty acids, amino acids, organic acids, benzenoids, and phenylpropanoic acids. The Z score represents the number of standard deviations from the mean. The metabolites are listed to the right of the heatmap. (E) Pathway analysis showed that differential fecal metabolites were enriched in the pathways, including phenylalanine metabolism, butanoate metabolism, and propanoate metabolism (*P* = 0.000142, 0.025806, and 0.025806, respectively). The rest are the involved pathways without significant differences.

The identified fecal metabolites were classified as amino acids, fatty acids, bile acids, organic acids, benzenoids, phenylpropanoic acids, SCFAs, carbohydrates, indoles, carnitines, and others (Data set S2 and Fig. S2 in the supplemental material). The results of a principal-component analysis (PCA) showed that the overall fecal metabolite profiles differed in the two groups (Fig. 4A). The plot of an orthogonal partial least-squares discriminant analysis (OPLS-DA) revealed that the composition of fecal metabolites changed significantly after the 6-week prednisone treatment (Fig. 4B). The changed fecal metabolites included SCFAs, fatty acids, amino acids, organic acids, benzenoids, and phenylpropanoic acids (Fig. 4C and D). In total, 11 downregulated and 10 upregulated fecal metabolites were identified based on a volcano plot and a variable importance in projection (VIP) analysis (Fig. 4D, Fig. S3, and Fig. S4). As shown in Table 1, the decreased metabolites included valeric acid, propanoic acid, isobutyric acid, isovaleric acid, caproic acid, hydrocinnamic acid, 2-phenylpropionate, phenylacetic acid, ortho-hydroxyphenylacetic acid, acetoacetic acid, and ethylmethylacetic acid. On the contrary, phenyllactic acid (PLA), hydroxyphenyllactic acid (OH-PLA), homovanillic acid, m-aminobenzoic acid, malonic acid, succinic acid, methylmalonic acid, 2-hydroxy-3-methybutyric acid, l-tryptophan, and l-phenylalanine (PAH) increased significantly. Pathway analysis showed that differential fecal metabolites were enriched in the pathways, including those for phenylalanine metabolism, butanoate metabolism, and propanoate metabolism (*P* = 0.000142, 0.025806, and 0.025806, respectively). The involved pathways without a significant difference included those for phenylalanine, tyrosine, and tryptophan biosynthesis; the synthesis and degradation of ketone bodies; valine, leucine, and isoleucine degradation; tyrosine metabolism, aminoacyl-tRNA biosynthesis; citrate cycle (TCA cycle); alanine, aspartate, and glutamate metabolism; and tryptophan metabolism (Fig. 4E).

**TABLE 1:** Profile of fecal metabolites in rats after 6-week prednisone treatment^*a*^

Class	Metabolite	CON	PRED	*P* value
SCFAs	Valeric acid	28.1333 ± 7.0798	14.2940 ± 4.1317	0.0013
	Propanoic acid	22.7566 ± 4.4820	14.2891 ± 4.9201	0.0057
	Isobutyric acid	7.4810 ± 3.5346	2.9502 ± 1.5464	0.0140
	Isovaleric acid	1.9924 ± 0.5135	1.2237 ± 0.7155	0.0175
	Caproic acid	1.1045 ± 0.5233	0.5190 ± 0.3999	0.0379
Phenylpropanoic acids	Hydrocinnamic acid	1.2233 ± 0.3337	0.5228 ± 0.1715	0.0008
	2-Phenylpropionate	1.1960 ± 0.3175	0.5227 ± 0.1724	0.0007
	Phenyllactic acid	0.0073 ± 0.0089	0.0357 ± 0.0365	0.0262
	Hydroxyphenyllactic acid	0.0051 ± 0.0013	0.0142 ± 0.0097	0.0379
Benzenoids	Phenylacetic acid	1.4107 ± 0.5759	0.8644 ± 0.4648	0.0262
	Ortho-hydroxyphenylacetic acid	0.0028 ± 0.0004	0.0021 ± 0.0004	0.0158
	Homovanillic acid	0.0063 ± 0.0013	0.0128 ± 0.0062	0.0151
	m-Aminobenzoic acid	0.0002 ± 0.0002	0.0004 ± 0.0002	0.0495
Organic acids	Acetoacetic acid	8.7525 ± 4.1035	3.8692 ± 3.0756	0.0283
	Malonic acid	0.0024 ± 0.0009	0.0147 ± 0.0103	0.0213
	Succinic acid	0.0731 ± 0.0527	1.8185 ± 1.4589	0.0262
Fatty acids	Ethylmethylacetic acid	1.8629 ± 0.7450	0.9630 ± 0.6738	0.0262
	Methylmalonic acid	0.1356 ± 0.1168	3.6283 ± 2.8569	0.0262
	2-Hydroxy-3-methylbutyric acid	0.0096 ± 0.0135	0.0874 ± 0.0881	0.0070
Amino acids	l-Phenylalanine	0.7265 ± 0.1550	0.9887 ± 0.2590	0.0448
	l-Tryptophan	0.0333 ± 0.0230	0.0630 ± 0.0188	0.0219

a CON, control group; PRED, prednisone group.

### Correlation between the relative abundance of gut microbiota and fecal metabolites in rats after long-term prednisone treatment.

**FIG 5:**
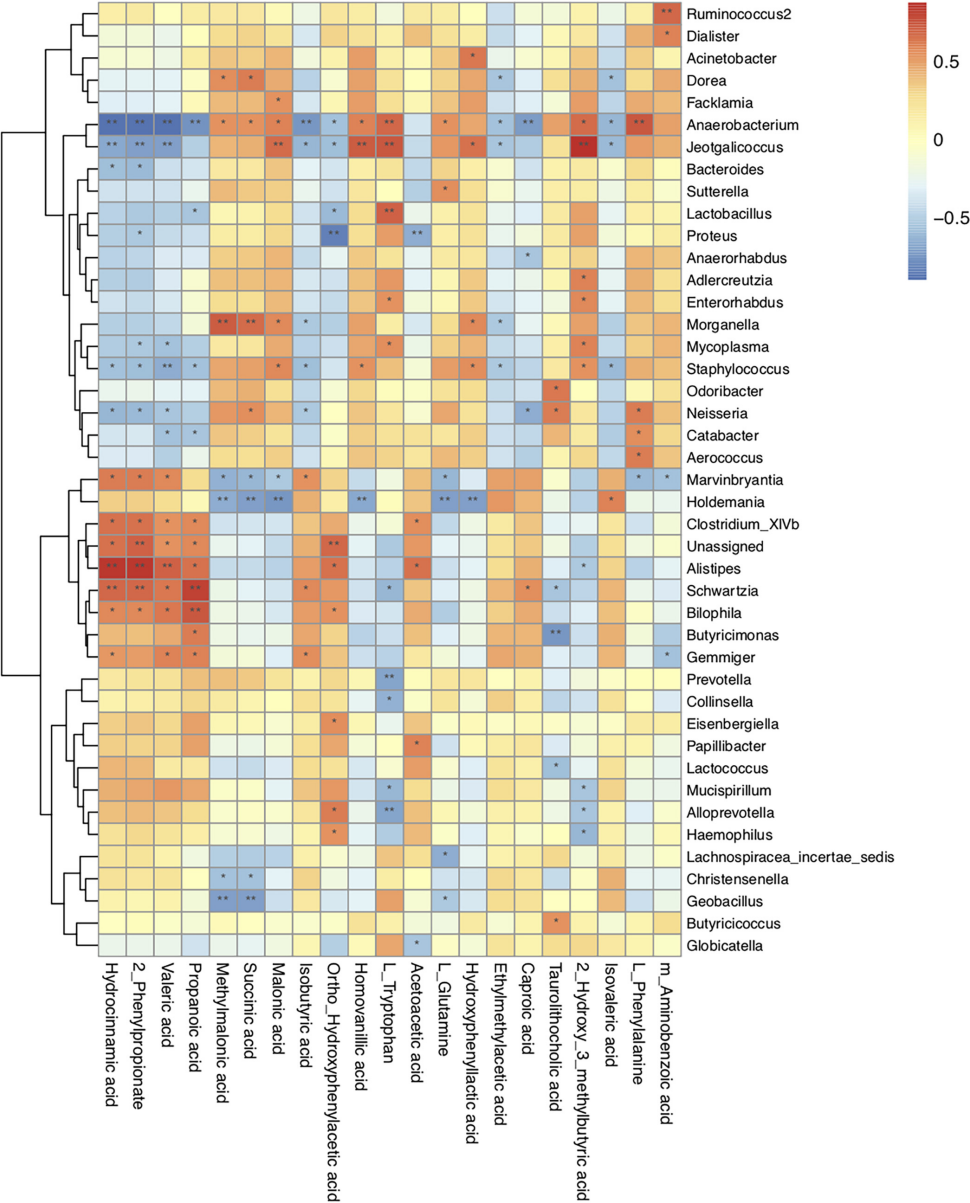
Correlation between the relative abundances of the microbial genera and fecal metabolites in rats after prednisone treatment. The Spearman correlation coefficient between the gut microbiota at the genus level and fecal metabolites in rats after the 6-week prednisone treatment was analyzed statistically. The data are shown in a heatmap. The red and blue represent positive and negative correlations, respectively. The degree of association is indicated by the intensity of the colors. *, P < 0.05; **, *P < *0.01.

In an attempt to explore the possible sources of the gut metabolites, we analyzed the correlation between the gut microbiota and fecal metabolites at the genus level (Fig. 5). Our analysis revealed that the change in abundance of *Eisenbergiella* spp. after prednisone treatment was positively correlated with an increase in ortho-hydroxyphenylacetic acid. The genus *Alistipes* was positively correlated with hydrocinnamic acid, 2-phenylpropionate, valeric acid, propanoic acid, ortho-hydroxyphenylacetic acid, and acetoacetic acid but negatively with 2-hydroxy-3-methybutyric acid. *Clostridium* XIVb was positively correlated with hydrocinnamic acid, 2-phenylpropionate, valeric acid, propanoic acid, and acetoacetic acid. The relative abundance of *Anaerobacterium* spp. was negatively associated with hydrocinnamic acid, 2-phenylpropionate, valeric acid, propanoic acid, isobutyric acid, ortho-hydroxyphenylacetic acid, ethylmethylacetic acid, caproic acid, and isovaleric acid. Finally, it was positively correlated with methylmalonic acid, succinic acid, malonic acid, homovanillic acid, l-tryptophan, l-glutamine, 2-hydroxy-3-methybutyric acid, and l-phenylalanine.

## DISCUSSION

Long-term prednisone therapy is widely used in immunological and rheumatic diseases. The anti-inflammatory and immunosuppressive effects of prednisone have been established, but its side effects compromise its application. The gut microbiota shapes the host immune system and metabolism. It is known that medication is capable of altering the gut flora. In the present study, our findings first showed that the composition of the gut microbiota and its metabolites was changed after long-term prednisone treatment in rats. Meanwhile, this is the first study to analyze the correlation between the gut microbiota and its metabolites in prednisone-treated rats.

Significant changes were not found in the microbial richness and diversity after the 6-week prednisone treatment in rats. Similar findings were also reported in children with primary nephrotic syndrome after a 4-week initial therapy, of which prednisone is a major medication (17). This may suggest that the overall ecosystem of the gut microbiota is stable after 4 to 6 weeks of prednisone treatment. However, as shown in this study, the composition of the gut microbial community differs significantly in rats exposed to prednisone therapy versus those without prednisone therapy. The relative abundances of the genera *Eisenbergiella*, *Alistipes*, and *Clostridium XIVb* decreased. It has been reported that *Eisenbergiella* is positively correlated with weight gain and fasting blood glucose levels in patients with gestational diabetes mellitus (18, 19). On the contrary, *Alistipes* is associated with lower weight gain during pregnancy (19). Taken together, the balance of *Eisenbergiella* and *Alistipes* may affect body weight after prednisone treatment.

Additionally, a 5-week prednisone treatment by gavage in lupus mice leads to genera changes which are distinct from our findings in rats. This may be caused by different types of rodents, potentially carrying transgenic disease (14). Additionally, members of *Clostridium* XIVb are well-known producers of butyrate, which is one of the SCFAs promoting the generation of Treg cells in the colon. Thus, it is suggested that decreased SCFA-producing microbes such as *Clostridium* XIVb bacteria may be harmful to the host in the long term. Meanwhile, the role of increased abundance of *Anaerobacterium* after prednisone treatment remains unknown. Therefore, our finding suggests that prednisone could alter the composition of the gut microbiota, which affects host immunity indirectly.

The gut microbiota influences host immunity through the generation of microbial metabolites. In accordance with the alterations in the microbial community, the concentration of fecal metabolites also changed after the 6-week prednisone treatment in this study. Pathway analysis showed that differential fecal metabolites were enriched in the pathways, including phenylalanine metabolism, butanoate metabolism, and propanoate metabolism. Among these decreased metabolites, SCFAs are well-known microbial metabolites. They function as energy resources for colon cells and also shape the host immune system. They are defined as free fatty acids containing fewer than 6 carbons, including acetic acid, propionic acid, butyric acid, and valeric acid and their branched fatty acids, such as isobutyric acid and isovaleric acid. Our findings showed that the relative abundances of valeric acid, propanoic acid, isobutyric acid, isovaleric acid, and caproic acid decreased after prednisone treatment. It has been shown that propanoic acid and butyrate promote the differentiation of Treg cells through histone deacetylase inhibition (20). Hence, long-term prednisone treatment may affect immunologic homeostasis. In addition, caproic acid promotes the differentiation of proinflammatory TH1 and TH17 cells (21). Decreased caproic acid may inhibit inflammation. Furthermore, our data on the correlation between altered gut microbiota and fecal metabolites suggested that lower production of SCFAs was associated with the decreased relative abundances of the genera *Alistipes* and *Clostridium* XIVb and increased *Anaerobacterium*. Therefore, microbiota-derived SCFAs might be involved in the pharmacology of prednisone.

Many fecal metabolites identified in this study have been reported to be associated with brain function. Homovanillic acid (HVA) is a metabolite of dopamine. Stress increases serum HVA as well as dexamethasone administration in humans (22, 23). In addition, glucocorticoid treatment could cause a psychotic reaction. However, it is still unknown whether the increased fecal HVA after prednisone treatment observed in this study was due to dopamine metabolism or generated from gut microbiota solely. It would be worthwhile to study the influence of HVA in steroid-related psychiatry. Malonic acid induces mitochondrial dysfunction by inhibiting succinate dehydrogenase. As a consequence, it promotes the generation of superoxide radicals, apoptosis, and secondary excitotoxicity (24). Malonic acid is commonly used for establishing an animal model of Huntington’s disease (25). It is unclear whether it is harmful to the nervous system when patients are exposed to increased malonic acid after long-term prednisone treatment. Tryptophan is an essential amino acid for humans and supplied in the diet, particularly in protein-rich foods (26). The catabolites of tryptophan, including 5-hydroxytryptamine and indole derivatives, have positive effects on host immune homeostasis, gut-brain signaling, and intestinal permeability (27, 28). The mostly thoroughly studied role of tryptophan is in brain physiology. Reduced circulating tryptophan was found in depressed patients (29). Tryptophan supplementation alleviated chronic insomnia (30). Increased tryptophan after prednisone treatment in rats suggested that gut microbiota could produce this essential amino acid. Moreover, it may be associated with psychiatric changes in some patients who receive long-term prednisone treatment. Additionally, methylmalonic acid is considered to be an oxidative stress inducer which promotes oxidative injury and suppresses antioxidant defenses. It is able to cause DNA damage in the rat brain and kidney (31). Hence, the increased methylmalonic acid after prednisone treatment may be detrimental to neurological and renal function. Taken together, these altered fecal metabolites might contribute to the psychiatric adverse events of prednisone.

Hydrocinnamic acid belongs to the phenylpropanoic acid group. Cisplatin-induced renal toxicity could be alleviated by manuka honey, of which hydrocinnamic acid is a key ingredient. It has been revealed that hydrocinnamic acid inhibits inflammation and oxidative stress (32). Additionally, it exhibits antifungal activity (33). Thus, anti-inflammatory effects might be diminished by decreased hydrocinnamic acid after long-term prednisone treatment. Phenylacetic acid (PAA) is the most abundant phenolic metabolite in human stool and has been identified as a uremic toxin in patients with end-stage renal disease (34, 35). It is associated with a prooxidant and proinflammatory status (35). Macrophage function could be impaired by PAA due to the inhibition of inducible nitric oxide synthases (36). Hence, prednisone might alleviate inflammation by decreasing PAA production.

Succinic acid is synthesized by the host and gut microbiota. It is not only an intermediary metabolite of the tricarboxylic acid cycle in the host but also is produced by the fermentation of carbohydrates in the gut microbiota (37). Increased succinate has been reported in dysbiosis of the intestinal flora (38). Elevated circulating succinate was also reported in obese individuals as caused by the disturbance of succinate-producing and -consuming microbiota (39). Succinate is involved in protein post-translational modification. Additionally, it serves as a proinflammatory mediator and is involved in cell stress (37, 40). Succinate can activate immune cells by binding to its receptor, succinate receptor 1 (SUCNR1) (41). We found that fecal succinate accumulated in rats after prednisone treatment. However, it needs to be verified whether increased fecal succinate is associated with steroid-related adverse events, particularly in obesity. In addition, fecal phenyllactic acid (PLA) and hydroxyphenyllactic acid (OH-PLA) are mainly produced by *Lactobacillus* spp. Both show anti-antifungal effects (42, 43). l-Phenylalanine (PAH) is an essential amino acid. Our data showed that 6-week prednisone treatment promotes the production of PLA, OH-PLA, and PAH. Therefore, it suggests that long-term glucocorticoid treatment may also be beneficial to the host via the gut microbiota.

In conclusion, long-term prednisone therapy altered the composition of the gut microbiota and the profile of fecal metabolites in rats. Fecal SCFAs might be inhibited by the decreased relative abundances of the genera *Alistipes* and *Clostridium* XIVb and increased abundance of *Anaerobacterium*. Our data might help to understand the pharmacology of prednisone in the aspect of the gut-kidney axis. However, there are some limitations in this study. It would be meaningful to measure the serum profile of the fecal metabolites, as it would help to verify the direct effects of these changes to the gut microbiota and metabolites on host. Furthermore, in view of the fact that prednisone is so widely used in immune disorders, the impacts of these changed factors on host immunity need to be verified in future studies.

## MATERIALS AND METHODS

### Animals and experimental design.

Animal experiments were performed in accordance with the policies and procedures of the Ethical Committee of the Shanghai Children’s Hospital. Specific-pathogen-free grade male Sprague-Dawley (SD) rats were purchased from Jiesijie Experimental Animal Company (Shanghai, China) at 5 weeks of age. All SD rats were housed under standard laboratory conditions (food and water *ad libitum*, 22 to 25°C, 50 to 60% relative humidity, 12:12-h light/dark cycle). After 10 days of habituation, 14 rats were randomly assigned to the control (CON) and prednisone (PRED) groups. Prednisone (Xinyi Pharmacy Company, Shanghai, China) at a dosage of 10 mg/kg body weight was given to rats daily for 6 weeks by gavage as described previously (44, 45). Rats in the CON group received an equivalent amount of water. Fresh fecal samples were collected from the rats after 6 weeks of treatment and stored at −80°C until use.

### 16S rRNA sequencing and analysis.

The methods of bacterial DNA extraction and sequencing were the same as described in our previous report (17). Total bacterial DNA was extracted from the fecal samples using the QIAamp DNA stool minikit (Qiagen, Hilden, Germany). The universal forward primer (5′-CCTACGGGNGGCWGCAG-3′) and reverse primer (5′-GACTACHVGGGTATCTAATCC-3′) were used for amplifying the V3 to V4 regions of the bacterial 16S rRNA gene (46). The PCR amplification reaction was performed in triplicate in a total volume of 25 μl. The reaction mixture consisted of 2.5 μl 10× PCR buffer II, 0.5 units of Herculase II DNA polymerase high fidelity (Agilent, USA), 0.4 μM of each primer, and 10 ng of template DNA. The following thermal cycling conditions were used: initial denaturation at 95°C for 2 min, 25 cycles of denaturation at 95°C for 20 s, primer annealing at 55°C for 20 s, extension at 72°C for 45 s, and a final extension at 72°C for 3 min. The 16S rRNA gene amplicon was sequenced on the Illumina MiSeq platform at Genesky Biotechnologies, Inc. (Shanghai, China). The qualified reads were clustered into operational taxonomic units (OTUs) using the UPARSE pipeline, based on a cutoff value of 97% sequence similarity. Alpha diversities, including the Chao1, ACE, Shannon, Simpson, and coverage indices, were calculated using Mothur software. The beta diversity analysis consisted of a principal-coordinate analysis (PCoA) and nonmetric multidimensional scaling (NMDS). R software v3.3.1 (vegan package) was used in PCoA and NMDS analysis based on the Bray-Curtis distance, Jaccard, and unweighted and weighted UniFrac metrics. The compositional alteration of the gut microbiota at different taxonomic levels was analyzed using the Metastats method. Bioinformatics analyses of the microbial data were performed by Genesky Biotechnologies, Inc.

### Fecal metabolomics and analysis.

In total, 10 mg of each fecal sample and 25 μl deionized water were homogenized with zirconium oxide beads for 3 min. Then, the sample was centrifuged (18,000 × g, 20 min) after mixing with 185 μl acetonitrile/methanol (8:2). The supernatant (20 μl) was transferred into 96-well plates, and 20 μl freshly prepared derivative reagent was subsequently added to each well. The plate was sealed and the derivatization was performed on a Biomek 4000 workstation (Beckman Coulter, Inc., CA, USA) at 30°C for 1 h. Then, the sample was diluted with an ice-cold 50% methanol solution (350 μl). The plate was centrifuged at 4°C (4,000 × g, 30 min) after placing at −20°C for 20 min. Finally, supernatant (135 μl) was transferred to a new 96-well plate with 15 μl internal standards in each well for liquid chromatography-mass spectrometry (LC-MS) analysis.

The fecal metabolites were quantitated in an ultra-performance liquid chromatography (UPLC) system coupled to a tandem mass spectrometry system (ACQUITY UPLC-Xevo TQ-S; Waters Corp., Milford, MA, USA). The ACQUITY UPLC ethylene bridged hybrid (BEH) C18 1.7-μm VanGuard precolumn (2.1 × 5 mm) and ACQUITY UPLC BEH C18 1.7-μm analytical column (2.1 × 100 mm) were used. Peptide mixture solution was added to a reverse phase trap column (Thermo Scientific Acclaim PepMap 100, 100 μm × 2 cm, nanoViper C18) linked to the C18 reversed-phase analytical column (Thermo Scientific Easy column, 10 cm long, 75 μm inner diameter, 3 μm resin) in buffer A (0.1% formic acid). It was separated with a gradient of 5% buffer B (acetonitrile/isopropyl alcohol [IPA] [90:10]) (0 to 1 min), 5 to 80% buffer B (1 to 12 min), 80 to 95% buffer B (12 to 15 min), 95 to 100% buffer B (15 to 16 min), 100% buffer B (16 to 18 min), 100 to 5% buffer B (18 to 18.1 min), and 5% buffer B (18.1 to 20 min) at a flow rate of 300 nl/min. The fecal metabolites were measured at Metabo-Profile Biotechnology Co. Ltd. (Shanghai, China). The population distribution of all fecal samples was evaluated using principal-component analysis (PCA). The differences between two groups were analyzed by orthogonal partial least-squares discriminant analysis (OPLS-DA). Identification of differential metabolites was based on a volcano plot (*P < *0.05) and VIP analysis (VIP > 1.0, *P < *0.05). The Spearman correlation coefficient between the gut microbiota and fecal metabolites was analyzed by Genesky Biotechnologies, Inc.

### Statistical analysis.

The rat body weight data were expressed as the mean ± standard deviation (SD). A nonparametric T-test was performed for comparison of the two groups using Prism v5.0 software (GraphPad, San Diego, CA, USA). A *P* value of <0.05 was considered to be statistically significant.

### Data availability.

The raw sequence data from the fecal microbiota in this paper were uploaded to the Genome Sequence Archive (GSA) (https://ngdc.cncb.ac.cn/gsa/) under accession number CRA004897. The original fecal metabolite data were also deposited in the MetaboLights database (https://www.ebi.ac.uk/metabolights/) under accession number MTBLS3414.
