# Supplementary material for: Integrative Analysis of Gut Microbiota and Fecal Metabolites in Rats after Prednisone Treatment
Source: Microbiol Spectr. 2021 Nov 10;9(3):e00650-21. doi: 10.1128/Spectrum.00650-21 (PMC8579919; doi:10.1128/Spectrum.00650-21)
Supplement: SUPPLEMENTAL FILE 1 — Supplemental material. Download SPECTRUM00650-21_Supp_1_seq10.pdf, PDF file, 0.3 MB [file spectrum00650-21_supp_1_seq10.pdf]

## Supplemental Materials For Publication

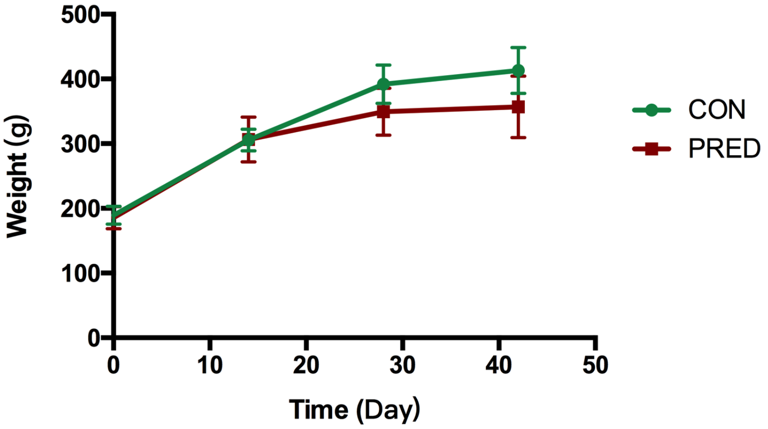


**Figure S1 The body weight of rats with and without 6-week prednisone treatment.** There is no significant difference between CON and PRED groups. *p*>0.05. Abbreviations: CON, control group. PRED, prednisone group.

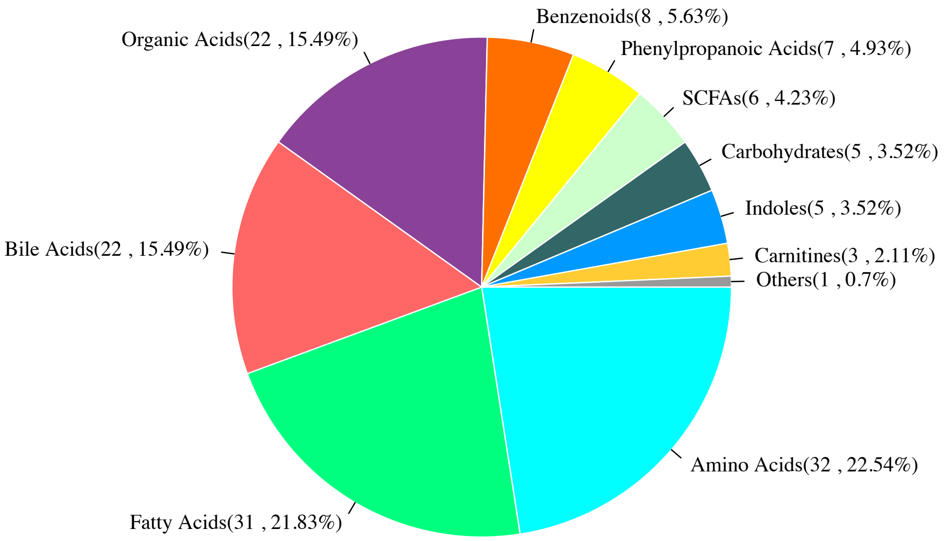


**Figure S2 The classification of identified fecal metabolites.** They were classified as amino acids, fatty acids, bile acids, organic acids, benzenoid, phenylpropanoic acids, SCFAs, carbohydrates, indoles, carnitines and others.

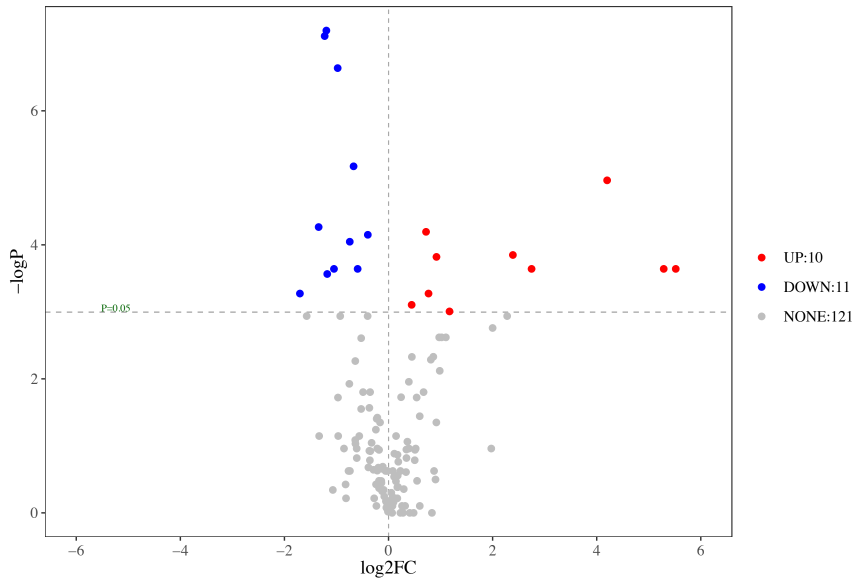


**Figure S3** Volcano plot analysis (*P*<0.05) were used for identifying differential fecal metabolites. Totally 11 downregulated and 10 upregulated fecal metabolites were identified.

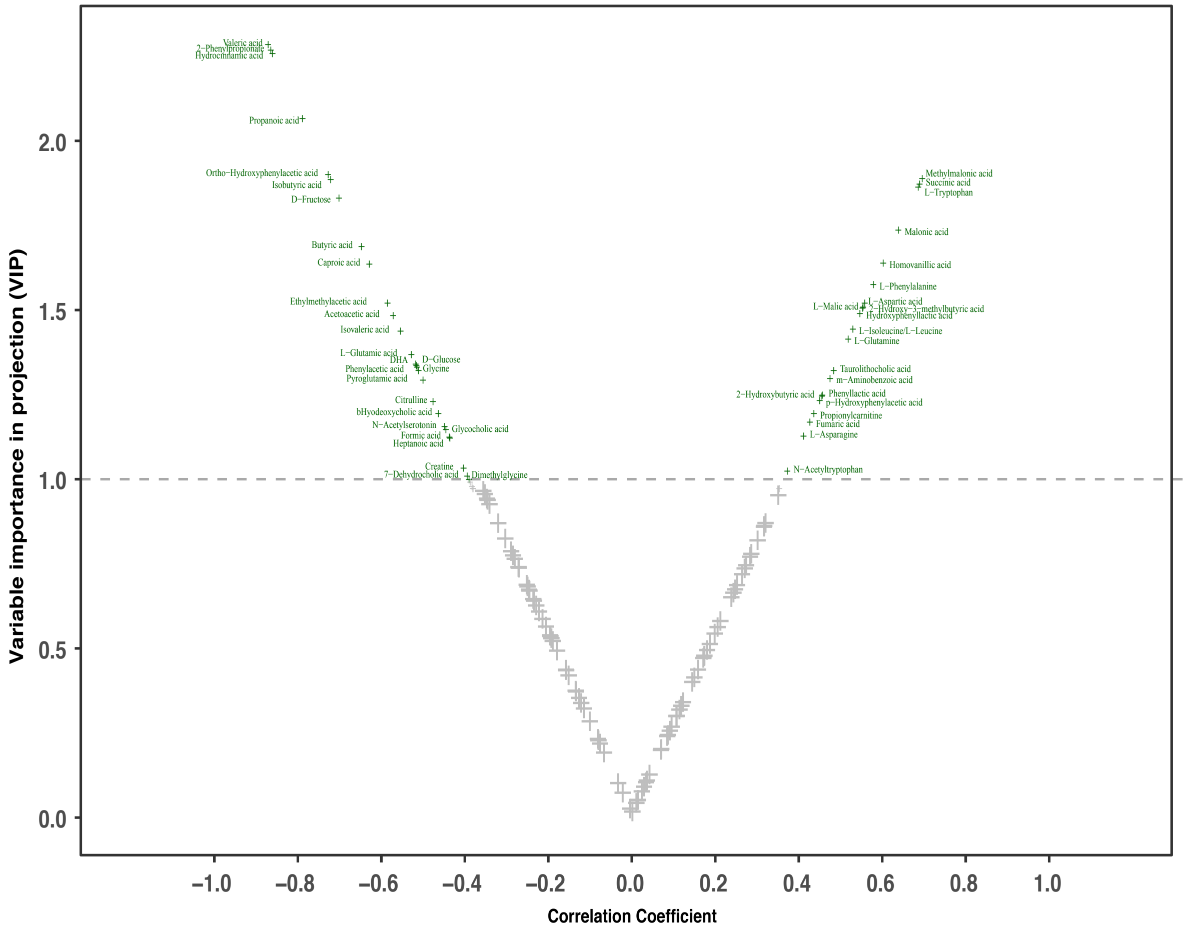


**Figure S4** VIP values>1 and *P* values <0.05 were the threshold parameters for selecting differential fecal metabolites.

**Supplementary material-1** The detailed information of identified OTUs in rats. Totally 2044 OTUs were detected in rats after 6-week prednisone treatment. Abbreviations: CON, control group. PRED, prednisone group.

**Supplementary material-2** The detected fecal metabolites in rats with and without prednisone treatment. Abbreviations: CON, control group. PRED, prednisone group.
